# Intrinsic Rivalry. Can White Bears Help Us With the Other Side of Consciousness?

**DOI:** 10.3389/fpsyg.2019.01087

**Published:** 2019-05-10

**Authors:** Marek Havlík, Eva Kozáková, Jiří Horáček

**Affiliations:** ^1^ National Institute of Mental Health, Klecany, Czechia; ^2^ Department of Psychology, Faculty of Arts, Charles University, Prague, Czechia; ^3^ Third Faculty of Medicine, Charles University, Prague, Czechia

**Keywords:** consciousness, perceptual consciousness, rivalry paradigms, intrinsic rivalry, intrusive thought

## Abstract

Studies of consciousness have traditionally been based mainly upon the perceptual domains of consciousness. However, there is another side of consciousness, represented by various types of intrinsic conscious experiences. Even though intrinsic experiences can represent up to 50% of our conscious experiences, they are still largely neglected in conscious studies. We assume there are two reasons for this. First, the field of intrinsic conscious experiences is methodologically far more problematic than any other. Second, specific paradigms for capturing the correlates of intrinsic conscious experiences are almost nonexistent. Nevertheless, we expect the intrinsic side of consciousness to soon take its place in conscious studies, but first new experimental paradigms will have to be devised, which would be of a similar design to the paradigms used in studies of perceptual consciousness. In this *hypothesis and theory* article, we propose such a hypothetical paradigm, presenting the exploratory data of our proof-of-concept study, discussing its use, and addressing its shortcomings and their possible remediation.

## Introduction

After the paradigm shift, when the long rejected topic of *consciousness* was accepted among popular empirical studies of the mind, potential candidates for neural correlates of consciousness (NCC) began to emerge, such as *gamma synchronous oscillations* (Crick and Koch, 1990; Summerfield et al., 2002; Doesburg et al., 2009; Gaillard et al., 2009; Melloni and Singer, 2010; Steinmann et al., 2014), *event-related oscillations of P300* (Babiloni et al., 2006; Del Cul et al., 2007; Lamy et al., 2009; Dehaene and Changeux, 2011; Salti et al., 2012; King et al., 2014), and activity of *thalamo-cortical circuits* (Newman and Baars, 1993; Bogen, 1995).

Many of these were used as the foundations for proposed theories of consciousness, such as the *Global Neural Workspace theory* (Dehaene and Naccache, 2001; Dehaene and Changeux, 2011; Dehaene et al., 2014), *Integrated Information theory* (Tononi, 2008), *Attended Intermediate Representation theory* (Prinz, 2005, 2012), the theory of *Thalamo-cortical Reentrant Loops* (Edelman, 1989; Edelman and Gally, 2013), *Recurrent Processing theory* (Lamme, 2006, 2010), *Higher Order Theories of Consciousness* (Lau and Rosenthal, 2011), and the recently proposed theory of the *Posterior Cortical Hot Zone* (Koch et al., 2016) and *First*-*order Representationalism* (Mehta and Mashour, 2013).

Some of these theories share some common elements but what really connects them is the fact that none of them has been universally accepted. What is shared, however, by most of the empirical scientists of consciousness, is the *core* method of conscious studies.

### The “Core” of Conscious Studies

The core approach to consciousness studies is the comparative method or comparative analysis (Koch, 2004) aiming at capturing the important difference between conscious and unconscious processing and thus identifying the neural processes responsible for conscious experience. However, this method is now being criticized from the position that genuine NCCs can be confounded with unconscious neural prerequisites and/or consequences of consciousness (Aru et al., 2012; Aru and Bachmann, 2015), which precede and follow conscious experiences. Nevertheless, it is reasonable to expect that comparative analysis will remain the core method of consciousness studies, unless a paradigm shift happens that would completely redefine our methods and experimental paradigms for finding NCCs.

Throughout the years, various paradigms for studying consciousness have been introduced. For example, *pattern illusion paradigms* represented by the well-known Kanisza triangle (Harris et al., 2011), *afterimages, phosphenes*, and other kinds of visual illusions (Kirschfeld, 1999), various *attention paradigms* such as the well-known *gorilla experiment* (Simons and Chabris, 1999), the *change-blindness paradigm,* where participants do not notice the change in the visual stimuli (Simons and Rensink, 2005), the *attentional blink paradigm* (Raymond et al., 1992) and *masking paradigms,* where participants fail to detect a second salient stimulus, which occurs soon after the first one, thus preventing it to enter the consciousness (Bachmann and Francis, 2013).

However, if there is a methodological *trademark* of conscious studies, it must be *multistable paradigms* based on the key feature of *rivalry*.

### Rivalry as a “Trademark” of Conscious Studies

**Figure 1:**
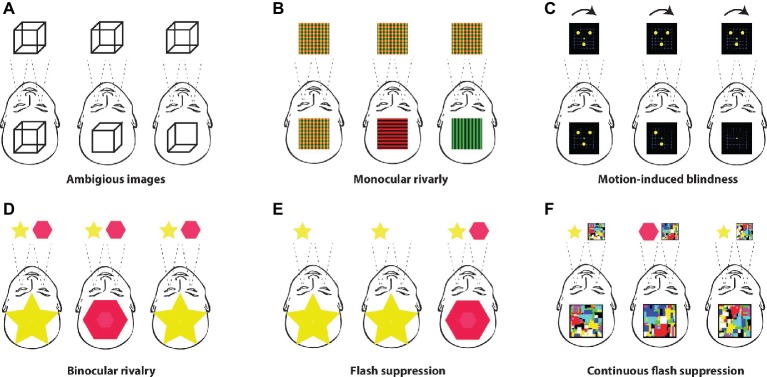
Multistable paradigms. **(A)** The first in the family of multistable paradigms are ambiguous images such as Necker cube, Rubin’s vase, and Schröder’s stairs. Prolonged viewing of such stable and unchanging images will eventually result in two different conscious perceptions, which spontaneously fluctuate between each other (e.g., Kleinschmidt et al., 1998; Schmack et al., 2015). **(B)** Monocular rivalry is a similar phenomenon. It occurs during prolonged viewing of two superimposed visual images, e.g., green and red lines. After some time, one image becomes clearer and eventually exclusively dominant, while the other fades from the conscious experience (O’Shea et al., 2009). **(C)** A similar form of fading away from consciousness can be achieved by the motion-induced blindness paradigm, in which several stable points, such as yellow circles, fade away from consciousness and are rendered unconscious when presented against moving dots (Bonneh et al., 2001). **(D)** Probably the best-known paradigm of conscious studies is binocular rivalry. In this visual paradigm, two different visual patterns (e.g., a star and a diamond, a face and a house, etc.) are presented to each eye separately. Conscious experience spontaneously fluctuates between the two images despite the constant and unchanging visual input to each eye (Klink et al., 2013). **(E)** The paradigm of flash suppression (Wilke et al., 2003) was developed as a methodological solution to the spontaneous and unpredictable switches of conscious perception under the binocular rivalry paradigm. A stimulus presented to one retina is rendered completely invisible by the sudden presentation (“flash”) of a different image to the other retina. **(F)** The continuous flash suppression paradigm utilizes constantly moving colored squares called “mondrians” (Tsuchiya and Koch, 2005). Such a salient and ever-changing stimulus presented to one eye completely prevents rivalrous stimuli presented to the other eye from entering the stream of consciousness, thus rendering them entirely invisible for long periods of time.

The term rivalry refers to the competition between underlying neural processes, in which the “winner takes all” and enters the stream of consciousness. *Ambiguous images,* such as the well-known Necker cube or Rubin’s face-vase, can be found within this family of visual paradigms (Figure 1). *Monocular rivalry* (O’Shea et al., 2009) is where one of two superimposed images becomes dominant over the other, *motion-induced blindness* (Bonneh et al., 2001) is where stimuli fade from conscious experience when presented against moving dots, and *binocular rivalry* (Klink et al., 2013) is where two different stimuli are presented to each retina, separately. In the *flash suppression* (Wilke et al., 2003) paradigm, the stimulus is rendered unconscious by another stimulus, which is presented in a “flash” to the other eye, and in *continuous flash suppression* (Tsuchiya and Koch, 2005), the static stimulus presented to one eye is constantly prevented from entering the stream of consciousness by to the second salient stimulus, which is usually rapidly changing.

These paradigms are based on the limits of perceptual processing demonstrated using the example of the most favored philosophical animal, the duck-rabbit. This visual illusion (which belongs among the paradigms of ambiguous images) represents a duck and a rabbit within a single picture. However, only the rabbit or the duck can be seen exclusively at one time but never together at the same time. The stimuli are not changing, but the conscious experience spontaneously fluctuates between two perceptual interpretations. The same goes for other multistable paradigms based on rivalry. During the prolonged viewing of multistable stimuli, the observer’s awareness changes between different states, where one is conscious, while the other is rendered unconscious (or it never enters the stream of consciousness, as in the case of continuous flash suppression). This is very important for the core method of consciousness studies based on a comparison of neuronal responses to these two neural representations.

Human beings are highly visual animals, so it is natural that most conscious paradigms are based on visual stimuli and visual processing, which is well documented. Nevertheless, it cannot be assumed that exclusively studying the visual domain of consciousness can bring universal conclusions about consciousness and conscious experience. That is why other perceptual domains of consciousness, such as auditory and olfactory consciousness, are now getting more attention.

There are reports that the most prominent correlates of visual consciousness, P300 and high gamma oscillations, also accompany auditory consciousness (e.g., Steinmann et al., 2014; Dykstra et al., 2016), olfactory consciousness (e.g., Mori et al., 2013), audiovisual perception (Balz et al., 2016), and tactile stimulation only when it is consciously perceived (Meador et al., 2002).

Even though such studies use various paradigms (for a review of various paradigms of auditory consciousness see Dykstra et al., 2017), a dominant category based again on rivalry starts to emerge. For example, studies of auditory consciousness use the so-called *dichotic listening task* (e.g., Brancucci et al., 2011; Brancucci and Tommasi, 2011; Steinmann et al., 2014), in which two auditory stimuli are projected into each ear through a set of headphones, thus creating “*binaural rivalry*” (Brancucci and Tommasi, 2011). Under such conditions, only one of the auditory stimuli enters the consciousness. Also, similar processes have been recently documented in studies of the domain of olfactory consciousness, where olfactory rivalry, is induced when two different odorants are presented exclusively to each nostril (e.g., Gottfried, 2009; Zhou and Chen, 2009; Stevenson and Mahmut, 2013).

“Rivalry” is considered a feasible method for perceptual studies of consciousness. However, even though the perceptual side of consciousness, which is focused exclusively on the processing of external stimuli, is quite well established, valid, and methodologically feasible, there is another largely neglected side of consciousness – intrinsic conscious experiences.

### The Other Side of Conscious Experience

Intrinsic conscious experiences are the term that we use to denote various mental contents that are not directly caused by external stimulation. Interest in such mental states was reignited by the discovery of the default mode network (DMN) (Callard et al., 2013; Havlík, 2017). Based on the interesting and anomalous findings of Biswal (Biswal et al., 1995; Biswal, 2012) and Shulman (Shulman et al., 1997), the DMN (Raichle et al., 2001; Raichle and Snyder, 2007) was established as one of the most important brain networks with activity accompanied by reoccurring loops in loss of attention to the external environment (Reichle et al., 2010; Smilek et al., 2010; Smallwood et al., 2011; Grandchamp et al., 2014; Huette et al., 2016). This network caused an essential scientific revolution in cognitive neuroscience, as studied using fMRI (Havlík, 2017). Since the beginning, its activity has been correlated with self-referential mental contents (Gusnard and Raichle, 2001) and intrinsic conscious experiences that occur in times of rest and without stimulation from the external environment.

Intrinsic conscious experiences can range from self-referential thinking (Gusnard and Raichle, 2001) to remembering and imagining the future (Addis et al., 2007; Buckner and Carroll, 2007), goal-directed thoughts (Spreng et al., 2010) such as statements about others (social cognition), including theory of mind (Buckner et al., 2008; Mars et al., 2012; Nekovarova et al., 2014), mental time travel (Østby et al., 2012) and others, which have a form similar to imagery simulations with the first or third point of view (Christian et al., 2013) with phenomenology close to dreaming, but with the implicit awareness that the experiences are the products of one’s own mind (Occhionero and Cicogna, 2016).

**Figure 2:**
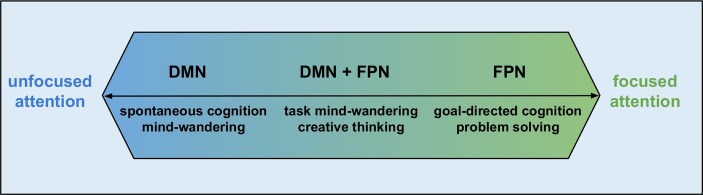
Attention and activity of DMN and FPN (inspired by Christoff et al., 2016).

Unfortunately, these intrinsic conscious experiences are now largely gathered under the umbrella term mind-wandering (Callard et al., 2013), which implicitly evokes the wrong intuition that all intrinsic conscious experiences are somehow passive. However, there are intrinsic experiences such as goal-directed thoughts, creative thinking, goal-directed imagination, etc., which clearly are not passive. This leads to the doubt of usefulness of the term mind-wandering, which can be documented by several studies (e.g., Christoff et al., 2018; Seli et al., 2018) that discuss how useful term mind-wandering really is, whether we should continue to use it or whether its use leads only to definitional haze and more confusion. Furthermore, this term is largely associated with the activity of DMN that further evokes the idea that all activities of DMN are related to passive and task-unrelated processing. Conclusions of several studies challenge this idea. The activity of DMN supports task-related cognition, such as active decisions (Andrews-Hanna et al., 2010) and level of details during working-memory (Sormaz et al., 2018). Contemporary views suggest DMN contribution to ongoing cognition that goes beyond the task-unrelated processing. However, these studies should not be taken as the proof that only DMN is responsible for all intrinsic experiences, passive or task-related. Gathering every intrinsic mental experience under the term “mind-wandering” and expecting activity of the DMN is serious misstep, which can also be documented by the emergence of hypothetical frameworks that try to better distinguish these intrinsic mental phenomena (Dixon et al., 2014; Christoff et al., 2016).However, there are some intrinsic states, which are also detached from the external environment but differ from spontaneous cognition in the degree of attention and are not accompanied solely by the activity of the DMN, but networks more directly associated with attention, such as the fronto-parietal network (Figure 2).

Based on large-scale surveys, various intrinsic experiences can constitute up to 50% of our daily cognition (Killingsworth and Gilbert, 2010; Song and Wang, 2012). This represents a substantial part of subjective experience, which is however still highly neglected in conscious studies and will be necessary for the universal or all-encompassing theory of consciousness.

Unfortunately, from a methodological point of view, this domain of consciousness is far more problematic than any other. Intrinsic conscious experiences have very few evident behavioral markers, which leads to substantial experimental pitfalls. For example, there is no adequate reporting procedure that would prevent reporting bias from disrupting ongoing intrinsic conscious experiences. However, the main reason why this domain of conscious experience has not been included among the topics of consciousness studies is certainly the lack of a specific method or paradigm required by conscious studies.

This does not mean that research of intrinsic experiences is completely without paradigms. For example, Hurlburt proposed a method called descriptive experience sampling (Hurlburt and Akhter, 2006). This method is based on collecting self-reports of participants’ ongoing experiences at the times when a beeper, which the participants carry, produces a sound at random. Of course, there are other methods (see Smallwood and Schooler, 2015 for review), such as the probe-caught method (which is very similar to Hurlburt’s version), where participants are randomly interrupted during the experiment and are asked (probed) about the exact content of their experience. Usually, they are asked whether they were focused on the task or whether they were mind-wandering. Other paradigms are the *open-ended method* and *the retrospective method*, which are applied immediately after the end of an experiment and rely on a questionnaire or on asking the participants directly about the content of their experiences during the scanning session of the experiment.

These methods can help to identify the specific mental states that occur in the stream of conscious experience and could certainly be considered as legitimate candidates for finding *neural correlates of specific states of consciousness* (see Chalmers, 2000), but unfortunately nothing more. Even though they can be relevant in exploring and finding the content of conscious experience, based on their design, they cannot say or report anything about the neural mechanisms that enable mental content to become conscious. Specifically, they do not meet the conditions that would be useful for a comparative method of conscious studies, such as multistable paradigms based on rivalry. Simply said, the above paradigms can only report on the content of conscious states, but they do not create rivalry conditions between them, which could reveal how the intrinsic mental states become conscious.

### Rivalry-Based Paradigm Between Intrinsic Experiences

The above text shows that “rivalry” can be considered as the *trademark* method for conscious studies. The question at hand is, “Would it be possible to develop the rivalry paradigm for studying intrinsic conscious experiences in a similar way to how it is used within the domains of visual (binocular), auditory (binaural) and olfactory consciousness?” We believe it would. Furthermore, the relevant paradigm (with such a rivalry condition) can be found in the pioneering work of Daniel M. Wegner on thought suppression and its paradoxical effects (Wegner et al., 1987; Wegner, 1989, 2011).

Inspired by Fyodor Dostoyevsky, Wegner in his experiment asked participants to verbalize their spontaneous stream of thought for 5 min. After this, the participants were invited to repeat the verbalization of thoughts with one additional condition:

“This time, try not to think of a white bear. Every time you say ‘white bear’ or have ‘white bear’ come to mind, though, please ring the bell on the table before you” (Wegner et al., 1987, p. 7).

Such intrusive thoughts were used to demonstrate the paradoxical or ironical effects of thought suppression, which says that suppression of thoughts is highly unproductive and leads only to the reoccurrence of such thoughts, preoccupation, and rumination (Wegner et al., 1987). Several other studies replicated Wegner’s conclusions, such as Lavy and Van den Hout (1990) using “vehicle” as intrusive thought instead of “white bear” and Clark et al. (1991, 1993) who used “green rabbit” as the intrusive thought.

**Figure 3:**
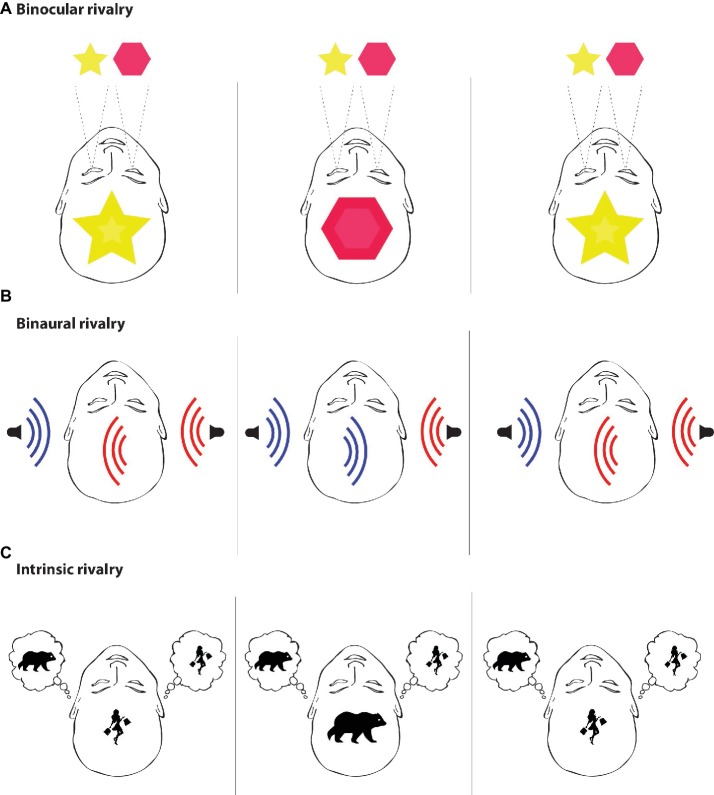
Rivalry. Rivalry paradigms are based on the limited capacity of neural processing. Put simply, two different mental phenomena cannot occur in the stream of consciousness at the same time. In experiments, two different stimuli are usually presented but only one of them becomes conscious. **(A)** Binocular rivalry is a visual paradigm in which two different visual patterns (e.g., a star and a diamond, a face and a house, etc.) are presented to each eye separately. The two images are not merged together and cannot be consciously perceived at the same time; therefore, conscious experience spontaneously fluctuates between the two images despite the constant and unchanging visual input. **(B)** Binaural rivalry is an auditory paradigm in which two different auditory stimuli are presented to each ear separately by a set of headphones and only one of them enters the stream of consciousness. The conditions guaranteeing the competition of the two auditory stimuli are known as the dichotic listening task, which is used for understanding hemispheric asymmetries (Brancucci et al., 2005). **(C)** Intrinsic rivalry is a possible paradigm aimed at intrinsic mental phenomena that occur during resting states. This paradigm is based on intrusive thoughts, such as the “white bear,” which constantly enter the stream of consciousness and render previously experienced content, such as thinking about “shopping,” unconscious. The paradigm of intrinsic rivalry is in a way similar to the rivalry conditions used in studies aimed at perceptual domains of consciousness, with the only difference that there is rivalry between two intrinsic mental states and not between two perceptual states. However, even in the case of intrinsic mental phenomena, two different mental states still cannot occur in the stream of consciousness at the same time. Therefore, intrusive thoughts can be used in future studies of consciousness as a paradigm aimed at neural correlates of intrinsic mental phenomena and may be quite useful for answering the question, “Under what neural conditions can intrinsic mental states become conscious?”

However, intrusive thoughts of this type have only been used within the scope of the paradoxical effects of thought suppression. We believe that similar simulation of intrusive thoughts in healthy volunteers under additional conditions could be eventually recruited by conscious studies as the specific paradigm for capturing the correlates of intrinsic conscious experiences. A closer look at the character of intrusive thoughts shows that they clearly have a *rivalrous* character toward the ongoing intrinsic conscious experiences, compete with them, and eventually “enter” the stream of consciousness. If an intrusive thought emerges and becomes conscious, it pushes other intrinsic conscious content out of the conscious spotlight, thus rendering it unconscious. This is similar to paradigms used in perceptual conscious studies, where two different stimuli cannot be perceived simultaneously, and conscious contents spontaneously change between each other. Hypothetically, the very same could be true for the other side of consciousness, where one cannot have two different thoughts at the same time (Figure 3).

## Data of Our Explorative Proof-of-Concept Study. Rivalry Between Intrinsic Mental Experiences

We developed a *rivalry* between two intrinsic mental experiences based on Wegner’s original design. We tested this method on 11 healthy participants (four males, mean age 28.4 years) with no history of neurological or mental disorder. The participants underwent a study using 3T functional magnetic resonance (fMRI).

### Methods

#### Design of the Experiment

The whole study consisted of two parts, each 10 min long. In the first part, the participants were given a task to have goal-directed thoughts, which were about their behavior in specific situations, such as shopping, taking a bus, ordering a meal at a restaurant, etc. In the second part, the participants were invited to reimagine their previous goal-directed thoughts with one additional condition. They were instructed not to think about a white bear while reimagining. A picture of a white bear was shown to the participants before entering MRI without any specific instructions.

This instruction (do not think about a white bear) paradoxically created two different goal-directed thoughts, which could not be conscious at the same time. The participants were, therefore, forced to undergo an experiment in which two intrinsic mental states continually competed for the conscious spotlight.

#### Report

Every time the white bear spontaneously entered the stream of consciousness and disrupted the goal-directed train of thought, the participants were obliged to press a button to mark the time in which the white bear entered their stream of consciousness and replaced the goal-directed thoughts. After reporting, the participants were instructed to go back to imagining the goal-directed scenarios and so on.

#### Statistical Analysis

Descriptive statistical analysis of target events (reporting of intrusive thoughts) was done using SPSS 2.

#### MRI

Magnetic resonance data acquisition was done at the National Institute of Mental Health, Klecany, using Siemens Prisma 3T. Echo Planar Imaging (EPI) sequences were used for functional scanning to obtain T2* weighted images with Blood Oxygenation Level-Dependent (BOLD) contrast consisting of 37 axial slices with TR = 2,000 ms, TE = 30 ms, flip angle = 70°, voxel size = 3 mm × 3 mm × 3 mm, with the resulting size of slice 64 voxels × 64 voxels. For anatomical localization and preprocessing 3D high-contrast, T1-weighted images were obtained using Magnetization Prepared Rapid Gradient Echo (MPRAGE) sequence with parameters of TR = 2,400 ms, TE = 2.34 ms, flip angle = 8°, voxel size = 0.7 mm × 0.7 mm × 0.7 mm.

Data were first preprocessed following standard procedure (i.e., realignment, normalization, slice-timing correction, smoothing with FWHM 8 mm) in SPM8, Statistical Parameter Mapping (Penny et al., 2011). The fMRI data analysis (event-related design) was based on behavioral reporting of button presses (signalization of the emergence of the intrusive thought) that created timestamps into a text file further used in the analysis. To exclude those possible cases when intrusive thoughts would be present rather continuously than as discrete events, we included only those events that were reported at least 5 s separate.

For a contrast analysis, we used the summary statistics approach (Monti, 2011). On the level of individual participants, contrasts of parameter estimates (i.e., percentual changes of BOLD signal) were calculated for each brain voxel between the conditions of the moment of reporting intrusive thoughts (events with the duration of 0 s) and the rest (contrast weights 1 vs. 0, respectively). Such a contrast shows us an activation linked to the emergence of intrusive thoughts exclusively.

The results from the single-subject analysis were used in the group-level analysis, where we used a one-sample *t*-test to test the hypothesis of null mean contrast in the whole group for each voxel. To correct for multiple testing, we used a standard method of Gaussian random fields that is implemented in SPM, so that FWER (Family-Wise Error Rate) was controlled at the level of 0.05.

### Main Findings. The fMRI Results

#### Behavioral Reports

The number of emergences of intrusive goal-directed thought was random during the experiment and the individual participants differed from each other in the rates of occurrence. The number of reports (events) ranged between 4 and 127, with an average value of 54.20 (SD = 41.58).

#### fMRI Results

**Figure 4:**
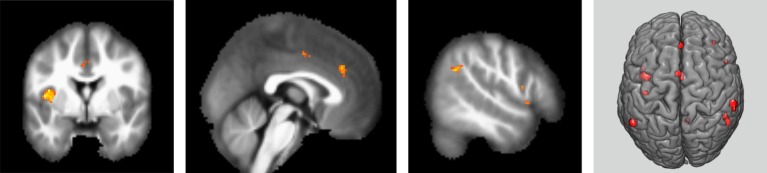
Areas of activation in contrast between intrusion versus goal-directed thoughts. *p* < 0.05 (FWE). T1-weighted MR image with highlighted areas of significant difference in hemodynamic response when emergence of intrusive thought was compared with goal-directed cognition (from left to right: left insula and dACC; left dACC; right angular and orbitofrontal gyrus; the same contrast displayed on 3D brain model). Minimal voxel size of the display area is five voxels, FWE-corrected voxel-wise threshold. Results of a group of 11 participants.

The results obtained based on the contrast between intrusive thought (white bear reports) and ongoing goal-directed experiences (imagining specific situations) or continuous presence of intrusive thoughts revealed neuronal increased BOLD activity in the left inferior parietal lobe (IPL), right fronto-parietal network (FPN) and both constituents of the salience network (SN), represented by the left insular cortex and the anterior cingulate cortex (*p* = 0.05 FWE, Figure 4). Using the opposite contrast (goal-directed scenarios versus intrusive thoughts) did not reveal any increase in activity at the specified level of significance (*p* = 0.05 FWE).

### Interpretation of Our Findings Within the Proposed Context

The main findings of this experiment were activations of the left IPL, right FPN, and SN. These findings are consistent with the results of several studies, which highlight and link the activity of these areas with moments of awareness and switches of attention toward a new stimulus (SN), executive control (FPN), and several conscious studies especially studies of binocular rivalry.

#### Inferior Parietal Cortex

Discussions of consciousness repeatedly draw attention to IPL lesions, which usually result in unilateral neglect (Vallar and Perani, 1986). Such patients begin to neglect the contralateral side to the lesion. Even though they have severe deficits in conscious experience, the contents of experience are not entirely lost. Marshall and Halligan (1988) in their experiment showed their patients a picture of two houses. One of them was on fire with visible flames on the neglected side. If asked whether the houses are the same, the patients answered that they are the same. However, when asked where they would prefer to live, the patients immediately chose the house that was not on fire. This is used by some as an argument that IPL can play an important role in mechanisms of conscious access, which are assumed to be located within post-sensory regions (Mehta and Mashour, 2013). Unilateral neglect is also considered as a disorder of attention (Prinz, 2012), which is largely considered as the necessary mechanism of consciousness (De Brigard and Prinz, 2010; Prinz, 2012).

Furthermore, the IPL/TPJ is considered as an important node in the ventral attention network (consisting of anterior insula and inferior frontal gyri) and is considered as a supramodal node, which accompanies vision, somatosensory processing, and audition (Corbetta et al., 2000, 2008; Downar et al., 2000; Corbetta and Shulman, 2002).

The IPL is considered to be involved in broad cognitive tasks, such as self-perception, social cognition, memory retrieval, and undirected thinking. However, due to the nature of our study, its functions within the scope of bottom-up attention and reorientation of attention (Igelström and Graziano, 2017) are the most interesting. The activity of the IPL correlates well with the reorientation of attention, particularly if the stimulus is unexpected but still relevant for the current task or behavior (Corbetta et al., 2000, 2008). The IPL also reacts to unexpected changes in sensory inputs, target detection, and brief stimulus-driven shifts in attention (Corbetta et al., 2000; Downar et al., 2000; Igelström and Graziano, 2017). In addition, the IPL is also one of the nodes of the FPN.

#### Fronto-Parietal Network

Our paradigm of intrinsic rivalry, activated FPN, is repeatedly reported to accompany the paradigm of binocular rivalry (Doesburg et al., 2009; Wilcke et al., 2009; Britz et al., 2011; Knapen et al., 2011; Roy et al., 2017). This similarity, of course, does not prove that intrinsic rivalry and binocular rivalry are analogous paradigms, but it suggests that intrinsic rivalry is not completely wrong.

Additionally, the FPN is almost identical to the fronto-parietal parts of the brain, which are proposed as central for the emergence of conscious experience within the Global Neural Workspace theory (Dehaene and Naccache, 2001; Dehaene and Changeux, 2011; Dehaene et al., 2014). The position of first-order representationalism (Mehta and Mashour, 2013) is similar, as it considers fronto-parietal regions as the post-sensory regions responsible for the entry of specific sensory content to consciousness.

Ventral FPN is active when the relevant stimulus appears outside of the focus of attention and will reorient attention toward this particular stimulus (Corbetta and Shulman, 2002; Trautwein et al., 2016). Ventral FPN activity also occurs in situations where the registered stimulus is relevant in a given context, while only the novelty of the stimulus is not sufficient for ventral FPN activation (Cabeza et al., 2012). The activity of the FPN was also reported in cases of an unexpected occurrence of the target stimulus in visual system studies (Corbetta and Shulman, 2002), which was further expanded to other sensory domains, such as auditory and tactile domains (Downar et al., 2000). These findings suggest that ventral FPN may be one of the general mechanisms responsible for the detection of stimuli and the reorientation of attention across various sensory domains. In addition, our findings extend this possibility to another domain, the domain of intrinsic experiences (in our case the white bear).

As explained above, mind-wandering experiences are usually entangled with the activity of the DMN. However, DMN activity and mind-wandering are the strongest during states of tuning out or zoning out (Smallwood and Schooler, 2015), when one’s attention is not focused. Under these conditions, intrinsic conscious experiences can lay completely out of the spotlight of consciousness. However, in cases where attention is fully focused on intrinsic experiences, such as planning, creative thinking or other goal-directed thoughts, FPN activity is observed (Figure 2; Christoff et al., 2016) and these types of intrinsic experiences are in the conscious spotlight.

Hence, the FPN correlates well with the degree of attention, which again represents for some an analogous mechanism for content entering into the stream of consciousness (De Brigard and Prinz, 2010; Prinz, 2012).

#### Salience Network

Coactivity of the insula and anterior cingulate cortex has recently been given a great deal of attention. Together these regions constitute the so-called salience network (SN), which plays an important role in switching between the main brain networks, the FPN, and DMN (Menon, 2015). It is also responsible for the detection of salient events (Seeley et al., 2007; Corbetta et al., 2008), such as a gradually disappearing target stimulus (Vidal et al., 2014) and detection of deviant stimuli (oddball) (Menon, 2015). The insula is also connected to many cognitive functions, some of which are close to consciousness. For example, the insula is active during meta-consciousness, monitoring of intrinsic mental states, and creative thinking (Fox and Christoff, 2014).

Specifically, there have been consistent reports of dACC activity in times of conflict between goals and distractors (Shenhav et al., 2016). Hasenkamp et al. (2012) reported activity of the SN when participants noticed a distraction from the task being performed. The activity of the dACC was also reported during target responses and target detection processes in tasks in which there was a strong competition of possible responses (Veen and Carter, 2002). In addition, a meta-analysis performed by Wager and Barrett (2017) showed that the insula was active in tasks requiring cognitive control of attention.

### Summary

Together, our data suggest that activity of the IPL, FPN, and SN accompanies the entry of a rivalrous unwanted thought (white bear) to the stream of consciousness. The activity of these regions correlates well with attention, orientation and reorientation of attention, intrusions and detection of distractors. Based on the literature and our data, it can be hypothesized that the activity of the FPN and IPL could be considered as the neural correlate of conscious access.

Nevertheless, this conclusion should be taken with caution due to several facts. First, our exploratory study was made on a small number of participants. Second, it will be necessary to implement EEG, to define the exact moment of entry to the stream of consciousness and its oscillatory correlates (data analysis could be based on the methods used in Doesburg et al., 2009). Finally, there are several shortcomings to this form of intrinsic rivalry paradigm, which became apparent after the study and which are openly addressed below.

## Intrinsic Rivalry Paradigm. Shortcomings and their Remediation

Our aim was to provide a more fitting methodological paradigm to the developing field of “intrinsic thought”, which could be included among other paradigms of consciousness studies. However, during the analysis of our original data, we encountered several ideas that would severely improve the study.

### Isolating the Conscious Content (BEAR)

One of the problems revealed later in our study was the difficulty of isolating or tracing the specific conscious content, in our case the white bear. For example, observation of the activity of the inferior temporal gyrus might have been expected. On the other hand, the baseline condition in our model (thinking about given scenarios) contained maybe too much semantic processing that might have precluded such finding. A more detailed specification of the stimuli/contents could better differentiate between them. For example, a seminal study of Tong et al. (1998) demonstrated, among other things, that specific conscious content can be to some extent traceable within the neural activity. Using the paradigm of binocular rivalry, Tong et al. stimulated participants with images of houses and faces. Perceptual and conscious shifts from house to face led to an increase in BOLD in the fusiform face area (FFA) and a decrease in the parahippocampal place area (PPA) and vice versa. Such contrasts can be used as supporting evidence that participants truly saw specific conscious content and, thus, the study is not solely dependent on the subjective reports.

Another way of tracing content through neural activity is the steady-state visually evoked potential (SSVEP) (Jamison et al., 2015; Roy et al., 2017). Under the paradigm of binocular rivalry, two different stimuli are flickering at two distinct frequencies. EEG can then be used to isolate or track these specific frequencies and, thus, provide more objective measurements of the ongoing subjective experience.

The problem of our intrinsic paradigm is that intrinsic experiences cannot be “tagged” with specific frequencies and, to our knowledge, there is no part of the brain that would be significantly active for the white bears. Hence, our study could reveal the correlates of conscious access, but not the correlates of conscious content.

Subsequent studies will be based on similar design; however, instead of “white bear”, it is planned to use imagination of faces and houses (imagine this particular house and do not imagine this particular face). This replacement of “white bear” with faces and houses will be done due to several reasons. First, the imagination of faces and houses is supposed to activate the FFA and PPA in the same way as it is with direct visual stimulation (O’Craven and Kanwisher, 2000). Under these conditions, goal-directed imagination as well as intrusive imagination could be traced within neural activity more objectively by the activity of FFA or PPA. Second, the imagination of a specific face and imagination of a specific house as two exclusively different images should prevent any possible fusion of two intrinsic thoughts and should lead to more discrete imagery of both types of images. After MRI session of our exploratory study, several participants described that at the end of the experiment they were unable to completely prevent the fusion of two rivalrous thoughts. White bear would appear at the party or in the school bus or restaurant. Clearly, in the final stages of the experiment, several participants did not experience a complete rivalry between goal-directed thought and intrusive thought but occasional fusions of the two thoughts. In our updated paradigm, we want to prevent this by using two simple goal-directed imaginations of two exclusively different stimuli, faces and houses, where one will play a role of goal-directed imagery, while the second will play the role of goal-directed intrusive imagery. We believe that this updated version of our intrinsic rivalry paradigm will lead to much stable experimental conditions, and due to its attention demanding character, it will prevent occurrence of task-unrelated thoughts. Third, this updated paradigm will be somewhat similar to the paradigm of binocular rivalry but with mental imagery instead of visual stimulation. We want to perform the binocular rivalry experiment along with the intrinsic rivalry experiment for three reasons. First, several participants of our intrinsic rivalry study described that even though they focused their attention fully on the task (for example, imagining eating at the restaurant) the thought of the “white bear” was still lurking in the background. We believe that this could be similar to binocular rivalry fusions of two pictures that occur during perceptual alterations (before one of stimuli becomes dominant). Second, we further want to compare the MRI data of these two paradigms (binocular rivalry vs. intrinsic rivalry) and found out the specific differences within the activity of FPN. Third, dominance periods of a stimulus under perceptual/binocular rivalry paradigm follow Poisson distribution (e.g., Levelt, 1967; Vallen et al., 1997; Murata et al., 2004). We want to further find out whether our updated version of intrinsic rivalry (which will be similar to binocular rivalry) will also show the resemblance to Poisson distribution in alterations between two different imaginations. This possible finding would further validate the resemblance of the two types of rivalry – the visual and the intrinsic one.

Moreover, subsequent studies of intrinsic rivalry could also benefit from the use of multi-voxel pattern analysis (MVPA). This relatively recent method uses details of specific configurations (patterns) of neural activations corresponding to given stimuli or cognitive states (Norman et al., 2006). An algorithm classifier is trained with a subset of data to be able to distinguish what pattern corresponds to what stimulus. The classifier is then able to decode which stimulus is probably being presented or imagined. The advantage of MVPA is that reporting part can be omitted completely, which makes it a suitable method for rivalry paradigms or mind reading (Haynes and Rees, 2005, 2006). First, a classifier would be trained based on prompts evoking non-rivalrous mental contents (presentation/imagination of one stimulus). These would be decoded later on during the rivalry conditions. The valence (neutral or involuntary/negative) can be evoked by manipulating instructions so that the classifier is trained in conditions closely resembling the ones during rivalry. It would be also useful to compare training during non-rivalrous condition with and without reporting with decoding of rivalry conditions also with and without reporting to check how reporting changes the decoding ability and also to extract it from respective contrasts.

### Possible Complexity of the White Bear

Another problem that later became apparent was the specific form in which “white bear” entered the stream of consciousness. Even though we showed a picture of a white bear within his natural environment to the participants before the MRI session, during the session itself we had no control over the form the participants would use, whether the bear would have the form of mental visual imagery (a picture), abstract thought or inner speech.

This problem has its roots within the definitional murkiness of “mind-wandering” and the activity of the DMN. The DMN should not stand as a representative for all intrinsic experiences; it is simply a network, which correlates with spontaneous cognition with low-level attention. Goal-directed thinking is something else than mind-wandering, and visual imagining is something else than counting the taxes in the head.

Again, in subsequent studies, it would be advisable to isolate the possible form of contents (faces/houses) to goal-directed mental imagery, solely (imagine this particular face), thus preventing any other form of intrinsic experiences to distort the participant’s conscious content. This and the fact that participants will be invited to imagine new faces and new houses (we plan to introduce new images of faces and houses during the experiment) will also help to prevent the emergence of unrelated thoughts due to the fact that imagination of new and salient images will be a highly attention-demanding task. Task-unrelated thoughts would have to be captured by the specialized (third) report due to the validity of the experimental design; however, we believe that adding third report condition (one for imagery of face, second for intrusive imagery, third for task-unrelated thought) would be cognitively too demanding for participants. Therefore, we want to utilize new stimuli and imagery of new pictures during the experiment, which will be highly attention-demanding and thus will not allow the occurrence of the task-unrelated thoughts.

### Lack of Sufficient Reporting

The participants in our study reported the entry of the white bear into the stream of consciousness and then went back to the goal-directed thoughts. Therefore, only the entry of the bear within the consciousness was reported. There was no report of how long it stayed conscious, there was no report of returning to goal-directed thoughts, and there was no report of possible transition (perceptual transition is a term found in studies of binocular rivalry, which addresses the state when two stimuli intermingle before one of them becomes dominant).

This shortcoming could be easily remedied using two buttons, where one would be pushed and held for reporting the dominance of specific intrinsic content (e.g., face) until the transition between two intrinsic contents occurs. For reporting intrinsic transition (two contents intermingled), two buttons would be held down until one imagination becomes dominant. This would be also useful for times when the participant is not entirely sure what the content of their imagination is. However, when we tried using both buttons, the participants repeatedly showed their discomfort and difficulty with such complex reporting. Therefore, in the next study, a simple joystick will be tested, where pulling and holding the joystick toward the participant would report imagining a face, the default position would report the imaginary transition and pushing the joystick away from the participant would report imagining a house.

Moreover, considering the problem of reports,recently a heated debate occurred over whether no-report paradigms could reveal the genuine correlates of consciousness (Overgaard and Fazekas, 2016) since the report itself distorts the data and with it the neural correlates of consciousness. Some believe that combining reporting and no-reporting will eventually lead to more valid data and true correlates of consciousness (Tsuchiya et al., 2015; Overgaard and Fazekas, 2016). Although these discussions are intriguing and worries are justified, there is still no consensus on the form of no-report paradigm (not even in perceptual studies of consciousness). We now consider utilizing the eye tracker to capture various eye behaviors that could possibly be considered as the behavior/ocular correlates of conscious access – access intrusive imagery into the stream of consciousness. This however requires two experimental sessions – the first with reporting of entry of intrusive image into consciousness and the second one, which will be report-free.

### Variability of Behavioural Responses

The lack of experimenter control was also a problem. This is exemplified by the extreme variability in the amount of button presses recorded across the participants. The recorded range was between 4 and 127, which can bring doubt as to whether the intrinsic rivalry paradigm is sufficiently reliable to track the same mechanisms across the various participants. In the next study, more detailed instructions should standardize the low-pass threshold for entering this intrusive thought into the stream of consciousness.

## How Useful Can the Intrinsic Rivalry Paradigm Be?

Based on the literature and the design of our study, we interpret the data from the position of the activity of attentional mechanisms, which can be considered analogous to mechanisms of conscious access. There are two competing mental contents, i.e., goal-directed thoughts and the intrusive white bear, which intrusively enters the stream of consciousness and replaces the other mental states.

The intrinsic paradigm, originally devised and tested to enrich the existing paradigms of conscious studies, can possibly have additional uses. First, this paradigm could produce useful information about how much the specific task at hand is engaging based on the frequency of intrusive thoughts, where a high frequency of intrusions says that the task at hand is boring and otherwise, a low frequency says that the task is highly engaging. Second, this paradigm could also be understood as a model of obsessive–compulsive disorder (OCD) or rumination. These disorders suppress the ability to regulate internally generated thoughts. Our data of the activations of the SN and FPN correspond to the results of OCD studies that demonstrate changes in connectivity between these networks (Stern et al., 2012). This suggests that the intrinsic paradigm can be potentially used for simulating compulsive thoughts in healthy participants. Such data could be later used for a contrast with OCD or rumination patients, possibly leading to isolation of the neural inability to regulate internally generated thoughts.

## Conclusion

In this hypothesis and theory article, we introduced the development and various paradigms of conscious studies, which are almost exclusively focused on the domains of perceptual processing and rely on rivalry conditions between stimuli. We also highlighted the fact that conscious studies neglect the other side of consciousness represented by intrinsic conscious experiences, which are unfortunately gathered under the term mind-wandering, which indicates the activity of the DMN.

We accented the need for feasible paradigms for studying intrinsic mental experiences. In addition, we introduced an innovative approach to study intrinsic consciousness based on intrinsic rivalry. Our paradigm, inducing the rivalry between intrinsic mental experiences, is similar to the well-established rivalry paradigms used in studies of perceptual domains of consciousness.

We also identified and addressed several shortcomings of our approach together with proposals for how to remediate them in future studies. Intrusive thoughts such as *white bears*, *pink elephants*, *green rabbits*, and other strange creatures could significantly help us in the future with the intrinsic side of conscious experience. Including them will be the necessary steps toward a unifying theory of consciousness, which must include both sides of consciousness – the evoked side, represented by perceptual experiences, and the intrinsic side, represented by various intrinsic mental phenomena.

## Ethics Statement

This study was carried out in accordance with the recommendations of Ethical Commission of the National Institute of Mental Health with written informed consent from all subjects. All subjects gave written informed consent in accordance with the Declaration of Helsinki. The protocol was approved by the Ethical Commission of the National Institute of Mental Health.

## Author Contributions

MH and JH wrote the article, and EK evaluated data.

### Conflict of Interest Statement

The authors declare that the research was conducted in the absence of any commercial or financial relationships that could be construed as a potential conflict of interest.
